# The Association of Medical Superintendents of American Institutions for the Insane

**Published:** 1853-06

**Authors:** 


Correspondence of the Philadelphia North American and U. S. Gazette.

### THE ASSOCIATION OF MEDICAL SUPERINTENDENTS OF AMERICAN INSTITUTIONS FOR THE INSANE.

*Baltimore, May* 11*th,* 1853.

In October, 1844, a number of the medical gentlemen connected with the institutions for the insane, in different parts of the United States, assembled in Philadelphia, and formed the Association whose title is given above, and which now holds annual meetings in different parts of the country. The association now numbers among its members the chief medical officers of thirty-one respectable institutions for the insane in nearly every part of the United States and of the British Provinces, and has already held two meetings in Philadelphia, one in Washington City, one in Utica, N. Y., one in Boston, two in the city of New York, and is now holding its eighth in Baltimore.

Much good has already resulted from the members contributing the results of their very extended experience in mental diseases, and by the free discussion of all subjects connected with the welfare of the insane, and especially in reference to the construction, organization and general management of institutions for their treatment. Wherever the meetings have been held, the members of the Association have invariably visited and carefully examined all the institutions for the insane in the vicinity, and have thus become familiar, not only with their good qualities, but also with the defects which have sometimes existed, and which the influence of the Association has often been instrumental in removing. Various valuable essays have been read at the different meetings, and been published in the journals specially devoted to insanity, or in the other medical periodicals of the country; and the sentiments of the Association have of late been frequently quoted as authority, by various officers of the General and State governments, as well as others interested in the preparation and management of hospitals for the insane.

Since the formation of the Association, in 1844, it has had to mourn the death of some of the most distinguished of American Physicians who have devoted themselves to the treatment of the insane, and whose labors have been recognized wherever insanity has been studied, or those suffering from it been properly cared for. The names of Woodward, Brigham, Macdonald, and others, will take a place on the scroll of fame with those of Pinel, Tuke, Esquirol, Rush, and all the worthies whose benevolence and determined energy led to a new system of treating the insane, based upon reason, humanity, and the plainest principles of practical christianity.

This Association, whose history and objects have been briefly sketched above, commenced its 8th meeting, at the Eutaw House, Baltimore, on Tuesday, the 10th of May, 1853, at 10 o'clock A. M.

The meeting was called to order by Dr. Buttolph, of New Jersey, (the Secretary,) and in the temporary absence of the President and Vice President, on motion of Dr. Kirkbride, of Philadelphia, Dr. F. T. Stribling, of the Western Asylum of Virginia, was appointed President pro tem.

On motion of Dr. Fonerden, of Maryland, a committee to prepare and arrange business for the meeting was appointed by the chair: Drs. Fonerden, Kirkbride and Buttolph being the committee.

On motion of Dr. Kirkbride, the members were authorized to invite visitors to attend the sessions of the Association.

On motion of Dr. Tyler, of New Hampshire, a committee, consisting of Drs. Tyler, Nicholls and Curwen, were appointed to select a place and designate the time for the next meeting of the Association.

Dr. Kirkbride exhibited and explained the plans of the new Alabama Hospital for the Insane, prepared under his direction, and which building is now being erected at Tuscaloosa, under the supervision of Messrs. Sloan and Stewart, architects of Philadelphia.

Dr. Nicholls presented and described the drawings for the new United States Hospital for the Insane of the Army and Navy, and of the District of Columbia, authorized by an act of the last Congress, and which has been already commenced.

Dr. Nicholls is the Superintendent of this Hospital, and the drawings are from his sketches, by Thomas U. Walter, Esq., the Government Architect.

Dr. Stewart, President of the Maryland Hospital for the Insane, exhibited, and had described by Mr. Niernstein, the architect of the building, the plans for the new Maryland State Institution for the Insane, which has just been located on a fine farm a few miles from the city of Baltimore.

All these plans differ somewhat in their details, but are very similar in their general character, and exhibit very strikingly the immense progress that has been made in planning this very important class of public institutions. Each is intended to accommodate 250 patients, with the necessary officers and attendants.

It is quite safe to say that, if the plans are faithfully carried out, these three hospitals will be the best, and most convenient, as well as most truly economical, to be found in any country. The plans presented are believed to conform in every essential respect to the principles heretofore adopted by the Association, and which are now pretty generally regarded as authority on the subject.

Invitations were received and accepted to visit the site of the new State Hospital for Maryland, and the Mount Hope Asylum this afternoon, the Maryland Hospital on Thursday afternoon, and the site of the United States Hospital at Washington on Friday. Adjourned to meet at the site of the new hospital at 4 P. M.

In the afternoon the members of the Association visited the insane in the Baltimore Alms House, situated on a beautiful farm of more than 300 acres, and about two miles from the city. The general hospital appears to be well conducted, but the condition of the insane is a reproach to the age, and a foul blot on the noble and enlightened city and county to which it belongs. Here are seen some of the horrors of a past age—fellow beings, male and female, chained by the leg, and permanently secured in low, damp, underground apartments, that ought never to be inhabited by those afflicted with any disease,—or exposed like wild beasts under a shed in the small, cheerless yards. Surely such a state of things cannot long be permitted, and a visit by the Maryland Legislature ought to be sufficient to induce them to make any appropriation, or to pass any laws necessary to prevent such a state of things so near her beautiful and hospitable Capital, or in any section of the State.

The Association then proceeded in carriages, under the guidance of Dr. Stewart, President of the Board of Trustees, to visit the site of the new State Hospital, six miles from Baltimore, and were highly gratified with the good judgment which has been exercised in the selection of a spot on which it is proposed to erect the noble structure which is to contribute so essentially to the comfort of the afflicted, and the benefit of the whole community. There are one hundred and thirty acres of land in the tract, and the views from it are of peculiar beauty, embracing Baltimore, the Chesapeake bay, with its shipping, and various other objects of interest.

The following members, representing the various institutions mentioned, are present at the meeting, viz: Dr. Bell, of the McLean Asylum, near Boston; Dr. Ray, of the Butler Hospital, at Providence, R. I.; Dr. Tyler, of the N. H. Asylum at Concord; Dr. Walker, of the South Boston Hospital; Dr. Benedict, of the New York State Hospital at Utica; Dr. Brown, of the Bloomingdale Asylum, N. Y.; Dr. Bullock, of the King's County Hospital, near Brooklyn, N. Y.; Dr. Buttolph, of the N. J. Hospital at Trenton; Dr. Kirkbride, of the Pennsylvania Hospital for the Insane at Philadelphia; Dr. Worthington, of the Friends' Asylum at Frankford, Pa.; Dr. Curwen, of the Pennsylvania State Hospital at Harrisburg; Dr. Stewart, of the Philadelphia Hospital, Blockley; Dr. Fonerden, of the Maryland Hospital; Dr. Stokes, of Mount Hope Asylum, Md.; Dr. Nichols, of the U. S. Hospital, Washington City; Dr. Stribling, of the Western Asylum of Virginia; Dr. Jarvis, of the Private Institution of Dorchester, Mass.; Dr. Patterson, of the Indiana Hospital; Dr. Kendricks, of the Ohio Hospital at Columbus, and Dr. Smith, of the Missouri Hospital at Fulton. Dr. Bell is President of the

Association; Dr. Ray, Vice President; Dr. Buttolph, Secretary, and Dr. Kirkbride, Treasurer.

Several gentlemen connected with the management of Hospitals for the insane are also present by invitation of the Association.

*Evening Session—May* 10.

A paper on the Lincoln (English) Lunatic Asylum, and a discussion of some of the peculiar views there entertained, prepared by Dr. Galt, of the Eastern Asylum of Virginia, was read, and the principles contained in the paper were critically examined in the discussion which followed, and which was participated in by Drs. Kirkbride, Benedict, Stribling, Jarvis, Stewart and Bell; the sentiments of all these gentlemen being, on many points, entirely at variance with those of the writer.

Dr. Kirkbride read a paper on night watching, and the general care of the patients at night in Institutions for the Insane, strongly urging its importance, and recommending a material extension of the means commonly employed. The Association then adjourned till 9 A. M. to-morrow.

*Morning Session—May* 11.

The paper, read by Dr. Kirkbride last evening, led to an animated and very interesting discussion, participated in by Drs. Benedict, Smith, Stewart, Ray, Jarvis, Walker, Kendricks, Worthington, Brown, Curwen, Fonerden, Stribling, Stokes, Nicholls, Buttolph, Bell and Kirkbride. The customs of all the institutions represented, in reference to the care of their patients at night, were fully examined, and very general uniformity of sentiment seemed to prevail as to what was desirable, although there is considerable difference still existing in practice on some points connected with the subject.

Some observations made by Dr. Kendricks, of Ohio, relative to modes occasionally adopted to break up the bad habits of the insane, led to remarks by Drs. Bell, Kirkbride, Stribling, Jarvis and Benedict, in the strongest reprobation of the use of any means in our institutions which partook of the character of punishment. Although temporary benefit might undoubtedly sometimes result, it was believed by the speakers, that this could not be received as a justification of such practices.

Dr. Nicholls read a paper, prepared by Dr. Galt, on "Pledges by the Insane," which, after a brief discussion, was laid on the table.

Dr. Jarvis read a very elaborate and valuable paper on the effects of over or perverted action of the Brain or Nervous System on Mental Health, which was fully discussed by Drs. Kirkbride, Fonerden, Ray, Buttolph, Bell, Smith and Jarvis, in decided commendation of the principles of education and modes of life recommended. This discussion led to a reference to the phrenological doctrines of the agency of the brain in the action of the mind, on which point the members were found to entertain quite different sentiments. At 2 P. M. the Association adjourned to 3½ P. M., and then repaired to the Mount Hope Asylum, which the members were invited to visit yesterday. This institution is in the suburbs of Baltimore, but the extension of the city, and the opening of its streets will soon compel its removal to a more private location. It is under the entire control of the Sisters of Charity, Dr. Stokes, its excellent physician, having charge only of the medical department.

The Association was received in the kindest manner, and elegantly entertained by the Sisters and Dr. Stokes, who exhibited and explained the uses of every part of the establishment, which is kept in the neatest order; and the general appearance of the patients is creditable to all concerned in their care. After returning from Mount Hope, the Association re-assembled at the Eutaw House, when Dr. Ray read a valuable paper "on undescribed forms of acute cerebral disease," detailing a large number of cases, and skilfully analyzing the views of various writers on the subject. The discussion of this paper was deferred for the present. Adjourned till 9 A. M. to-morrow.

The members of the Asssociation accepted the invitation of Dr. R. S. Stewart, the indefatigable and veteran friend of the insane, and were elegantly entertained at his hospitable mansion during the evening.

*Morning Session—May* 12.

After the reading of the minutes, the Association proceeded to a discussion of the paper of Dr. Ray, on certain forms of cerebral disease, in which Drs. Kirkbride, Patterson, Walker, Kendrick, Stribling, Benedict, Brown, Ray and Bell participated. The form of disease referred to by Dr. Ray, seemed to differ from that heretofore described to the Association by Dr. Bell, and which, although quite prevalent in some districts, is entirely unknown in other sections of the United States.

On motion of Dr. Stribling, a Committee of finance, consisting of Drs. Stribling, Kirkbride and Buttolph were appointed.

Letters from Drs. Earle and Chandler, relative to reports from Hospitals, were read and laid on the table.

The Committee to select a place and proper time for the next meeting of the Association, recommended that it be held at the city of Washington, on the second Tuesday of May, 1854, at 10 o'clock A. M.

Dr. Ray moved to amend by substituting Providence, and fixing the time in 1855, but the amendments were lost, and the recommendation of the committee subsequently agreed to, with great unanimity.

Dr. Kirkbride, by appointment of the Association last year, read a series of propositions relative to the proper organization of Hospitals for the Insane, which, after mature consideration and a very full discussion, and various amendments at the afternoon and evening sessions, were unanimously adopted. The proper organization of Institutions for the Insane is one of the most important measures that can engage the attention of those getting up new Hospitals, or promoting the welfare and prosperity of the old; and the sentiments of the Association on the subject, the result, as they are, of long practical experience in every section of the country, and thus formally expressed, will, it is hoped, lead to important results.

Dr. Stewart, the President of the Board of Trustees of the Maryland Hospital, by invitation, explained the organization of that institution, and gave an interesting statement of his views on the whole subject.

